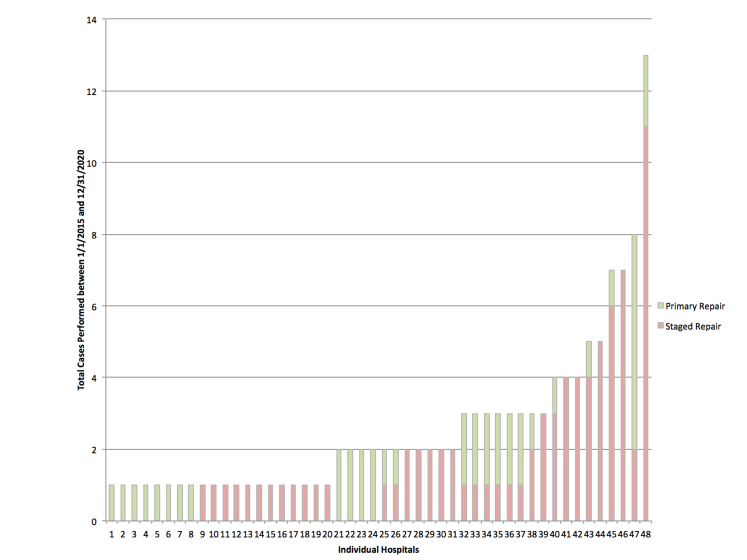# Primary vs Staged Biventricular Repair for Neonatal IAA with VSD and LVOTO

**DOI:** 10.1016/j.atssr.2024.04.025

**Published:** 2024-05-22

**Authors:** Joseph R. Nellis, Jacob C. Scherba, James M. Meza, Joseph W. Turek, Nicholas D. Andersen

**Affiliations:** 1 Division of Cardiothoracic Surgery, Department of Surgery, Duke University Hospital, Durham, North Carolina; 2 Duke University School of Medicine, Duke University, Durham, North Carolina

## Abstract

**Background:**

This study sought to determine the safety of primary and staged biventricular repair in neonates with interrupted aortic arch (IAA), ventricular septal defect (VSD), and severe left ventricular outflow tract obstruction (LVOTO).

**Methods:**

Patients with a fundamental diagnosis of IAA and VSD between 2015 and 2020 were extracted from The Society of Thoracic Surgeons National Database by using a Participant User File. The objective was to compare outcomes for neonates undergoing primary and staged Yasui and Ross operations. Primary end points were operative morbidity and mortality.

**Results:**

During the study period, 11.4% (123 of 1079) of neonates with a fundamental diagnosis of IAA and VSD underwent operations indicative of severe LVOTO. Of these patients, 42 (34%) underwent primary biventricular repair (Yasui or Ross/Ross-Konno), and 81 underwent a potential staging procedure (Norwood or hybrid stage I). No differences were observed in preoperative patient characteristics between groups. Neonates undergoing staged repair experienced fewer major complications (0 vs 1; *P* = .04) and total complications (2 vs 4; *P* = .02), but similar operative mortality (5% vs 12%; *P* = .27) as neonates undergoing primary repair. A total of 58 patients undergoing Rastelli, biventricular repair, Yasui, or Ross/Ross-Konno operations with a diagnosis of IAA and VSD and history of neonatal Norwood or hybrid stage I procedures were also identified. Operative mortality for second-stage biventricular conversion operations was 2% (1 of 58). Only 4 centers performed 1 or more complex biventricular repairs for IAA and VSD with LVOTO per year.

**Conclusions:**

Primary and staged biventricular repairs for IAA and VSD with LVOTO are associated with low operative mortality in the modern era and may be favorable to long-term single-ventricle palliation.

**In Short**

▪ Neonatal biventricular repair for IAA and VSD with severe LVOTO continues to be a rare condition.
▪ Neonates with IAA and VSD and severe LVOTO who are undergoing biventricular repair experience low operative mortality in the modern era.
▪ Neonates undergoing staged biventricular repair experience fewer complications.

Yasui and colleagues^1^ published the first description of a single-staged biventricular repair for neonatal interrupted aortic arch (IAA) with ventricular septal defect (VSD) and severe left ventricular outflow tract obstruction (LVOTO) in 1986. Although modifications have been made, biventricular repairs performed today for this diagnosis continue to be categorized as either primary or staged. Primary biventricular repairs include the Ross, Ross-Konno, and Yasui procedure (a Damus-Kaye-Stansel procedure, right ventricle–to–pulmonary artery conduit, and VSD repair to create a biventricular system). Alternatively, staged repairs use a single-ventricle Norwood or hybrid stage I repair followed by a delayed biventricular conversion (Rastelli/staged Yasui, Yasui, Ross/Ross-Konno) or long-term single-ventricle palliation.

Institutional and individual preferences continue to dictate the management of IAA and VSD with LVOTO. In a 2014 series by Shihata and colleagues,^2^ 24 neonates underwent staged biventricular repair with a 6-year survival of 86% for nonsyndromic patients. Similar results have been reported by Kanter and colleagues^3^ (6 primary repairs, 15 staged repairs; 75% 5-year actuarial survival), Nakano and colleagues^4^ (6 primary repairs, 11 staged; 87% survival), and Mallios and colleagues^5^ (3 primary, 32 staged; 91% survival). There has been a dearth of studies large enough to report meaningfully on differences in operative morbidity and mortality between primary and staged biventricular repairs; a 2010 Congenital Heart Surgeons’ Society series investigating the management of critical LVOTO included only 8 neonates who underwent a Yasui procedure.^6^

The Society of Thoracic Surgeons (STS) Congenital Heart Surgery Database (CHSD) is the largest congenital and pediatric heart surgery database in the world, with more than 600,000 validated entries to date.^7^ With the use of this database, the primary objective of this study was to identify differences in operative morbidity and mortality between primary and staged biventricular repairs for neonatal IAA and VSD with LVOTO in the modern era. Additional aims of the study included descriptive analysis of second-stage operations. Our goal was to gain a better understanding of the perioperative outcomes in this complex patient population.

## Patients and Methods

### Patient Populations

**Supplementary Figure 1:**
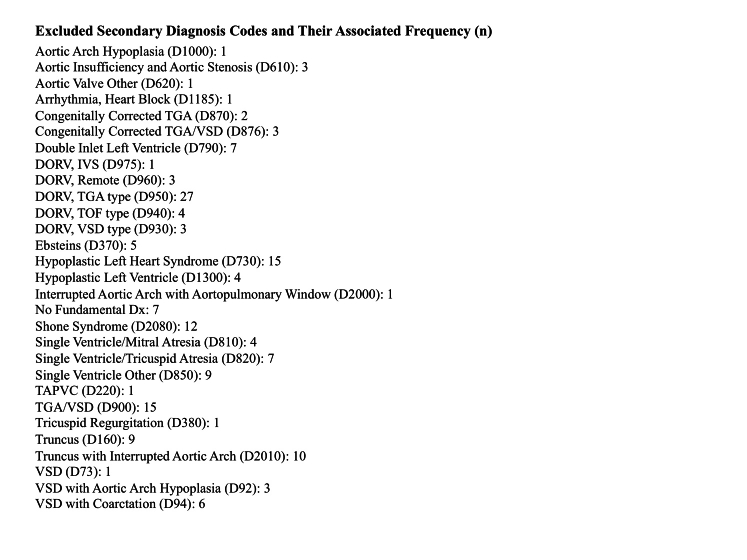


**Supplementary Figure 2:**
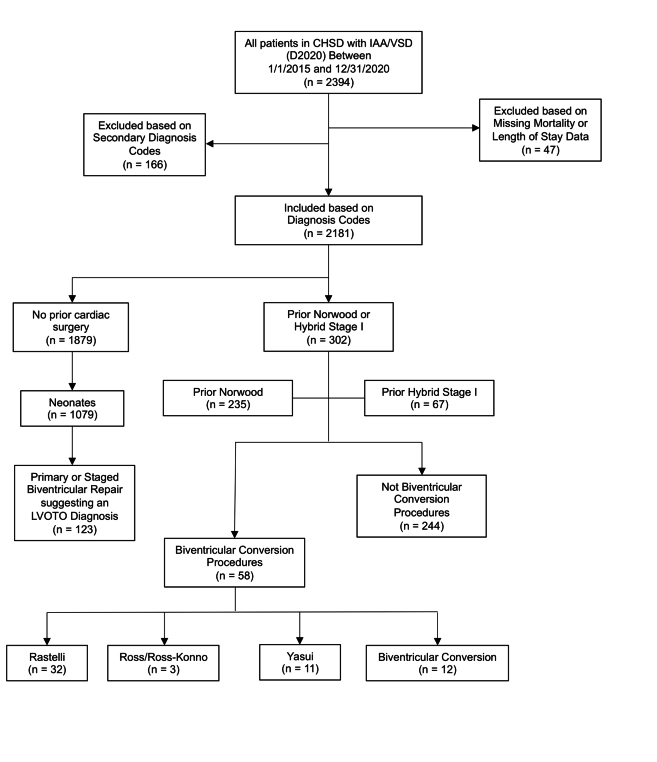


The data for this research were provided by the STS National Database Participant User File Research Program. Patients included in the STS CHSD with a fundamental diagnosis of IAA and VSD (D2020) between January 1, 2015 and December 31, 2020 were initially included. Patients with secondary diagnosis codes suggestive of alternative diagnoses, including single-ventricle anatomy, were excluded (Supplemental Figure 1). Patient cohorts were further defined on the basis of age at operation and history of previous cardiac surgery (Supplemental Figure 2). Briefly, neonates (aged <31 days) with a diagnosis of IAA and VSD and no previous cardiac operations who underwent Yasui (STS procedure code P2755), Ross/Ross-Konno (P740, P760), Norwood (P870), or hybrid stage I (P2180) operations represented the first patient cohort. The second patient cohort included patients with a diagnosis of IAA and VSD and a history of previous Norwood or hybrid stage I operation who underwent Rastelli (P1150), biventricular conversion (P880), Yasui (P2755), or Ross/Ross-Konno (P740, P760) operations. Patients excluded from analysis included those with missing mortality data (eg, mortality status at hospital discharge, mortality status at database discharge, or mortality status 30 days after surgery) or postoperative length of stay data.

### Variables

Variables provided by STS for this analysis included the following: patient demographics; fundamental diagnosis; any diagnosis of IAA and VSD; known preoperative risk factors; syndromes; chromosomal abnormalities; noncardiac abnormalities; hospital where patients’ surgery was performed; surgery year; history of Norwood or hybrid stage I operation; whether Norwood, hybrid stage I, Yasui, Ross/Ross-Konno, Rastelli, or biventricular conversion was performed; age and weight at the time of operation, cardiopulmonary bypass and cross clamp times; length of stay; postoperative length of stay; complications; and mortality. Within noncardiac abnormalities, airway issues were defined as the composite rate of laryngomalacia, tracheal stenosis, tracheomalacia, bronchomalacia, and abnormality of the trachea or bronchus. Major complications and operative mortality, as previously defined, were study end points.^8^ Patients experiencing operative mortality were excluded from length of stay calculations; and for this group, postoperative length of stay was recorded as postoperative time to mortality.

### Statistical Analysis

This is a descriptive study. Categoric values were reported as frequency and percentage of incidence (n, %) and were compared using the Fisher exact test. The distribution of continuous variables was tested for normality by using the Shapiro-Wilk test. Normally distributed data were reported as mean (SD), and skewed data were reported as median (interquartile range). Continuous variables were compared using Mann-Whitney *U* analysis. Data analysis was performed at the investigators’ institution with SPSS Statistics software version 27 (IBM Corp).

## Results

### Primary vs Staged Biventricular Repair for Neonatal IAA and VSD With LVOTO

Between January 1, 2015 and December 31, 2020, 1079 neonates with IAA and VSD and no previous cardiac surgical procedures were entered into the STS CHSD. A total of 123 (11%) of these patients underwent operations indicative of severe LVOTO. A total of 81 (66%) patients underwent a Norwood or hybrid stage I repair (staged repair), whereas 42 (34%) underwent a Yasui or Ross/Ross-Konno repair, suggesting a single-stage neonatal biventricular repair (Supplemental Table 1). There were no statistically significant differences in preoperative patient characteristics for neonates undergoing primary or staged repairs. Neonates undergoing staged repair had shorter median operative (261 minutes vs 342 minutes; *P* < .001), bypass (142 minutes vs 197 minutes; *P* = .02), and cross-clamp (59.5 minutes vs 128 minutes; *P* < .001) times than did neonates undergoing primary repair.

**Supplementary Figure 3:**
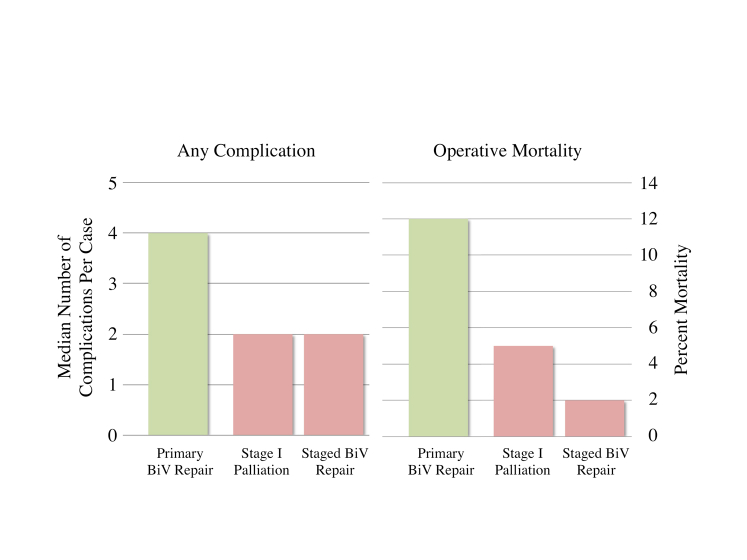


Complications were common after primary and staged repair (92% vs 83%; *P* = .26). Complications occurring in more than 10% of patients included recurrent laryngeal nerve injury (19%), postoperative low cardiac output (20%), arrhythmia requiring medical intervention (23%), unplanned cardiac operation (27%), and planned open sternum (48%). Major complications affected neonates who were undergoing primary repair more often (61% vs 36%; *P* = .02). Neonates undergoing primary repair experienced a higher number of total complications (4 vs 2; *P* = .02) (Supplemental Figure 3) and major complications (1 vs 0; *P* = .04) than did neonates undergoing staged repair. Moreover, although operative mortality was similar for primary and staged neonatal repair (12% vs 5%, respectively; *P* = .27) (Supplemental Figure 3), neonates undergoing primary biventricular repair experienced this end point much sooner than did neonates in the staged cohort (15 days vs 151 days; respectively; *P* = .02).

Of the 48 centers reporting cases during the study period, only 4 centers (8%) averaged at least 1 case per year (Supplemental Figure 4).

### Second-Stage Operations for Biventricular Repair of IAA and VSD With LVOTO

Between January 1, 2015 and December 31, 2020, 302 patients with IAA and VSD and a previous history of either a Norwood operation or a hybrid stage I operation were entered into the STS CHSD. Only 58 (19%) patients (median age, 8.7 months) underwent a secondary operation consistent with biventricular repair (Supplemental Table 2). The median operative, bypass, and cross-clamp times were 349 minutes (interquartile range, 293-454 minutes), 177 minutes (interquartile range, 145-223 minutes), and 110 minutes (interquartile range, 90-143 minutes), respectively. Patients incurred a median number of 2 (interquartile range, 1-4) total complications and 0 (interquartile range, 0-1) major complications. Operative mortality was 2% (Supplemental Figure 3).

## Comment

Repair options for newborns with IAA and VSD with severe LVOTO include primary biventricular repair, staged biventricular repair, and long-term single-ventricle palliation. In this report, we sought to assess the procedural safety of primary and staged biventricular repair as competing therapies to single-ventricle palliation. In a large series, we used the STS CHSD to assess differences in morbidity and mortality for 123 neonates with IAA and VSD with LVOTO who underwent primary and staged biventricular repair. Our results demonstrate that staged repairs were more common and were associated with fewer perioperative complications, but they carried similar overall mortality as primary biventricular repair. A lack of longitudinal data prevented true, paired, interstage analysis, although independent second-stage data suggest that delayed biventricular conversion is well tolerated and may be favorable to long-term single-ventricle palliation. 2, 3, 4, 5^,^^7^^,^^9^^,^^10^

Second-stage data do not suggest that operative mortality is shifted or postponed for patients undergoing staged biventricular repair of IAA and VSD with LVOTO. The operative mortality for patients with a history of IAA and VSD with LVOTO and previous Norwood or hybrid stage I repair who underwent completion biventricular repair was 2%, which is similar to the mortality for bidirectional Glenn operations at the national level and is much lower than the mortality for the comprehensive stage 2 procedure.^7^^,^^9^ However, less than 25% of patients with a history of Norwood or hybrid-Norwood repair underwent a second-stage procedure consistent with biventricular conversion, a finding suggesting that the majority of patients who had initial palliation with a Norwood or hybrid-Norwood operation remained on the single-ventricle pathway. Although some of these patients may have carried additional diagnoses (eg, ventricular or mitral valve hypoplasia or complex VSD anatomy) that prevented them from undergoing biventricular conversion, other patients may have never been offered biventricular repair because of individual provider concerns. Long-term analysis supports biventricular repair over single-ventricle palliation, when possible.^2^, ^3^, ^4^, ^5^^,^^7^^,^^9^^,^^10^

In our study, the Norwood operation, as the primary index procedure, was well tolerated. Neonates with IAA and VSD with LVOTO who underwent a Norwood operation experienced 6% operative mortality, and no patients who underwent second-stage biventricular repair with a history of Norwood operation experienced operative mortality. Although the Norwood operation has been associated with an operative mortality rate of 7% to 39% overall, patients with IAA and VSD with LVOTO did better in our limited analysis, perhaps given the stability of the 2-ventricle Norwood circulation.^10^ Our analysis also showed that neonatal operations for IAA and VSD with LVOTO are rare, with less than one-half of all STS CHSD participating programs reporting a neonatal operation for IAA and VSD with LVOTO repair during the 6-year study. These data suggest that in rare, infrequently encountered situations, it may be best to “do what you know” and proceed with a low-risk neonatal Norwood operation vs a complex neonatal biventricular repair. After initial stabilization with a Norwood operation, outpatient referral for evaluation of 2-ventricle candidacy at an experienced center could be considered at a later age, if experience or comfort with biventricular conversion is lacking at the primary program.

This study has several limitations. First is the lack of longitudinal data. The STS CHSD provides unparalleled granularity during the perioperative period, but for patients requiring staged operations, comprehensive analysis becomes extremely difficult. Without longitudinal data, we cannot reliably make conclusions on interstage attrition, the percentage of patients completing staged biventricular repair, the need for reoperation, or other time-matched comparisons between patients undergoing primary and staged repairs. Second is sample size. To detect a 50% difference in operative mortality between the 2 groups, assuming rates of 12% and 6%, the study would need to have had 356 patients in each arm. The diagnosis code for IAA and VSD (D2020) was introduced into the STS CHSD in 2015, thus limiting our ability to collect a larger cohort. Finally, patients with the diagnosis of IAA and VSD with mild LVOTO at 1 institution may have undergone a Yasui or staged procedure, whereas the same patient at another institution may have undergone aortic arch reconstruction and VSD closure. Without more granular anatomic data, we cannot speak to how the degree of LVOTO influences physician decision making for these patients.

In conclusion, formal analysis of infrequent although complex operations will always be hindered by sample size. In the case of biventricular repair of neonatal IAA and VSD with LVOTO, staged repair is associated with fewer major complications than primary repair (36% vs 61%, respectively; *P* = .02) and low operative mortality (5% vs 12%, respectively; *P* = .27) in the modern era.

## Figures and Tables

**Supplementary Figure 4:**