# Supplementary material for: Primary vs Staged Biventricular Repair for Neonatal IAA with VSD and LVOTO
Source: Ann Thorac Surg Short Rep. 2024 May 22;2(4):815–9. doi: 10.1016/j.atssr.2024.04.025 (PMC11708735; doi:10.1016/j.atssr.2024.04.025)
Supplement: Supplementary Figure Legend [file mmc1.docx]

**Supplemental Figure 1. Excluded Secondary Diagnosis Codes and Their Associated Frequency (n).** Abbreviations include double outlet right ventricle (DORV), intact ventricular septum (IVS), total anomalous pulmonary venous connection (TAPVC), transposition of the great arteries (TGA), tetralogy of Fallot (TOF), and ventricular septal defect (VSD).

**Supplemental Figure 2. Sequential Study Cohort Construction Based on Inclusion and Exclusion Criteria.** Abbreviations include Congenital Heart Surgery Database (CHSD), Interrupted Aortic Arch, Ventricular Septal Defect (IAA/VSD).

**Supplemental Figure 3. Median Morbidity and Mortality following Primary and Staged Biventricular Repair for IAA/VSD with Severe LVOTO.** Primary biventricular (BiV) repairs included Yasui and Ross/Ross-Konno. Initial stage 1 palliation included Norwood or hybrid stage 1 operation. Staged biventricular repairs included Rastelli, Yasui, Ross/Ross-Konno, and biventricular conversion. All patients undergoing primary biventricular repair or initial stage 1 palliation are neonates.

**Supplemental Figure 4. Number of Primary and Staged Neonatal Biventricular Repairs for IAA/VSD with Severe LVOTO by Hospital.** Hospitals in the STS CHSD reporting primary or staged neonatal biventricular repair for IAA/VSD between 2015 and 2020. Hospitals arranged sequentially in increasing hospital volume over the six year study period.
