# Supplementary material for: Primary vs Staged Biventricular Repair for Neonatal IAA with VSD and LVOTO
Source: Ann Thorac Surg Short Rep. 2024 May 22;2(4):815–9. doi: 10.1016/j.atssr.2024.04.025 (PMC11708735; doi:10.1016/j.atssr.2024.04.025)
Supplement: Supplementary Table 1 [file mmc2.docx]

| **Supplemental Table 1. Neonates with Fundamental Dx IAA/VSD Included in STS Congenital Heart Surgery Database 2015-2020** | | | | | | | | | | |
| --- | --- | --- | --- | --- | --- | --- | --- | --- | --- | --- |
|  | **All Patients** | **Patients without LVOTO** | **Patients with LVOTO** | | | | **Grouped by Approach** | | |  |
|  |  |  | **Yasui** | **Ross/Ross-Konno** | **Norwood** | **Hybrid Stage I** | **Primary** | **Staged** | **p-value** |  |
| **Number of Patients** | 1079 | 956 | 37 | 5 | 51 | 30 | 42 | 81 |  |  |
| **Gestation (weeks)** | 38.0 (2.6) | 38.0 (2.6) | 37.9 (1.9) | 38.2 (1.5) | 38 (1.1) | 37.6 (2.9) | 38 (1.9) | 37.9 (2) | 0.85 |  |
| **Prematurity (n/N, %)** | 126/1077 (12%) | 108/954 (11%) | 4/37 (11%) | 1/5 (20%) | 6/51 (12%) | 7/30 (23%) | 5/42 (12%) | 13/81 (16%) | 0.60 |  |
| **Birth Weight (kg)** | 2.9 (0.61) | 2.9 (0.61) | 2.9 (0.56) | 2.6 (0.5) | 3 (0.52) | 2.7 (0.5) | 2.9 (0.56) | 2.9 (0.56) | 0.94 |  |
| **Male (n/N, %)** | 553/1079 (51%) | 493/956 (52%) | 22/37 (59%) | 3/5 (60%) | 20/51 (39%) | 15/30 (50%) | 25/42 (60%) | 35/81 (43%) | 0.09 |  |
| **Caucasian (n/N, %)** | 678/1019 (67%) | 600/900 (67%) | 25/34 (74%) | 3/5 (60%) | 31/50 (62%) | 19/30 (63%) | 28/39 (72%) | 50/80 (63%) | 0.41 |  |
| **Preoperative Factors** |  |  |  |  |  |  |  |  |  |  |
| Shock, ongoing | 59/1053 (6%) | 55/932 (6%) | 0/36 (0%) | 1/5 (20%) | 3/50 (6%) | 0/30 (0%) | 1/41 (2%) | 3/80 (4%) | >0.99 |  |
| Shock, resolved | 166/1053 (16%) | 140/932 (15%) | 8/36 (22%) | 0/5 (0%) | 10/50 (20%) | 8/30 (27%) | 8/41 (20%) | 18/80 (23%) | 0.81 |  |
| Taking steroids | 105/1053 (10%) | 101/932 (11%) | 2/36 (6%) | 0/5 (0%) | 2/50 (4%) | 0/30 (0%) | 2/41 (5%) | 2/80 (3%) | 0.60 |  |
| Hypocoagulable | 110/1053 (10%) | 100/932 (11%) | 5/36 (14%) | 1/5 (20%) | 2/50 (4%) | 2/30 (7%) | 6/41 (15%) | 4/80 (5%) | 0.08 |  |
| Renal Dysfunction | 50/1053 (5%) | 36/932 (4%) | 2/36 (6%) | 0/5 (0%) | 3/50 (6%) | 9/30 (30%) | 2/41 (5%) | 12/80 (15%) | 0.13 |  |
| Renal Failure, dialysis | 19/1053 (2%) | 17/932 (2%) | 0/36 (0%) | 1/5 (20%) | 0/50 (0%) | 1/30 (3%) | 1/41 (2%) | 1/80 (1%) | >0.99 |  |
| Mechanical Ventilation | 503/1053 (48%) | 460/932 (49%) | 13/36 (36%) | 1/5 (20%) | 17/50 (34%) | 12/30 (40%) | 14/41 (34%) | 29/80 (36%) | 0.84 |  |
| Any | 715/1053 (68%) | 646/932 (69%) | 22/36 (61%) | 2/5 (40%) | 24/50 (48%) | 21/30 (70%) | 24/41 (59%) | 45/80 (56%) | 0.84 |  |
| **Syndromes** |  |  |  |  |  |  |  |  |  |  |
| DiGeorge | 445/923 (48%) | 396/826 (48%) | 19/33 (58%) | 1/2 (50%) | 24/45 (53%) | 5/17 (29%) | 20/35 (57%) | 29/62 (47%) | 0.39 |  |
| Fetal Alcohol Syn or Drug Exposure | 54/923 (6%) | 47/826 (6%) | 2/33 (6%) | 0/2 (0%) | 4/45 (9%) | 1/17 (6%) | 2/35 (6%) | 5/62 (8%) | >0.99 |  |
| Any | 502/923 (54%) | 444/826 (54%) | 20/33 (61%) | 1/2 (50%) | 29/45 (64%) | 8/17 (47%) | 21/35 (60%) | 37/62 (60%) | >0.99 |  |
| **Chromosomal Abnormalities** |  |  |  |  |  |  |  |  |  |  |
| 22q11 | 438/928 (47%) | 387/831 (47%) | 21/33 (64%) | 1/2 (50%) | 24/45 (53%) | 5/17 (29%) | 22/35 (63%) | 29/62 (47%) | 0.14 |  |
| Any | 523/928 (56%) | 462/831 (56%) | 22/33 (67%) | 1/2 (50%) | 28/45 (62%) | 10/17 (59%) | 23/35 (66%) | 38/62 (61%) | 0.82 |  |
| **Non-Cardiac Abnormalities** |  |  |  |  |  |  |  |  |  |  |
| Airway Issues | 123/906 (14%) | 106/812 (13%) | 9/33 (27%) | 0/2 (0%) | 6/44 (14%) | 2/15 (13%) | 9/35 (26%) | 8/59 (14%) | 0.16 |  |
| Any | 247/906 (27%) | 216/812 (27%) | 14/33 (42%) | 0/2 (0%) | 14/44 (32%) | 3/15 (20%) | 14/35 (40%) | 17/59 (29%) | 0.36 |  |
| **Perioperative Details** |  |  |  |  |  |  |  |  |  |  |
| Age (days) | 9 (6-14) | 9 (6-14) | 8 (5-9) | 7 (6-9) | 7 (5-10) | 7.5 (6-15.8) | 7.5 (5.7-9) | 7 (6-11) | 0.91 |  |
| Weight (kg) | 3.1 (1.6) | 3.1 (1.7) | 2.9 (0.5) | 2.9 (0.5) | 3.1 (0.5) | 2.8 (0.6) | 3.0 (0.5) | 3.0 (0.6) | 0.90 |  |
| Operative Time (min) | 225 (56-320) | 202 (50-309) | 344 (306-409) | 353 (278-525) | 335 (262-407) | 125 (91-151) | 342 (290-413) | 261 (149-359) | <0.001 |  |
| CPB Time (min) | 143 (109-179) | 140 (109-172) | 199 (169-227) | 237 (83-321) | 153 (108-192) | 0 (0) | 197 (165-228) | 142 (57-180) | 0.002 |  |
| Cross Clamp Time (min) | 74 (51-102) | 74 (51-99) | 128 (96-146) | 129 (34-143) | 64 (46-90) | 0 (0) | 128 (91-143) | 59.5 (1.7-82) | <0.001 |  |
| **Complications** |  |  |  |  |  |  |  |  |  |  |
| Major Complication | 261/1046 (25%) | 209/927 (23%) | 19/33 (58%) | 4/5 (80%) | 21/51 (41%) | 8/30 (27%) | 23/38 (61%) | 29/81 (36%) | 0.02 |  |
| Median Number of Major Complications per Patient | 0 (0-0) | 0 (0-0) | 1 (0-2) | 1 (1-1) | 0 (0-1) | 0 (0-0.8) | 1 (0-2) | 0 (0-1) | 0.04 |  |
| Ventilator > 7 days | 132/1046 (13%) | 93/927 (10%) | 12/33 (36%) | 2/5 (40%) | 18/51 (35%) | 7/30 (23%) | 14/38 (37%) | 25/81 (31%) | 0.53 |  |
| Unplanned Reintubation | 77/1046 (7%) | 56/927 (6%) | 7/33 (21%) | 0/5 (0%) | 9/51 (18%) | 5/30 (17%) | 7/38 (18%) | 14/81 (17%) | >0.99 |  |
| Recurrent Laryngeal Nerve Injury | 166/1046 (16%) | 143/927 (15%) | 8/33 (24%) | 1/5 (20%) | 12/51 (24%) | 2/30 (7%) | 9/38 (24%) | 14/81 (17%) | 0.45 |  |
| Any Complication | 598/1046 (57%) | 496/927 (54%) | 31/33 (94%) | 4/5 (80%) | 48/51 (94%) | 19/30 (63%) | 35/38 (92%) | 67/81 (83%) | 0.26 |  |
| Median Number of Complications per Patient | 1 (0-3) | 1 (0-3) | 4 (1-6) | 4 (4-5) | 3 (1-4) | 1 (0-2.8) | 4 (1-6) | 2 (1-4) | 0.02 |  |
| **Length of Stay (days)** | 34 (22-54) | 33 (21-52) | 43 (30-64) | 49 (46-56) | 36 (27-62) | 32 (25-67) | 44 (31-63) | 34 (26-66) | 0.22 |  |
| **Postoperative Length of Stay (days)** | 26 (15-43) | 25 (14-41) | 38 (25-55) | 43 (39-50) | 30 (22-52) | 24 (19-63) | 38 (25-56) | 29 (20-54) | 0.19 |  |
| **Operative Mortality** | 60/1079 (6%) | 51/956 (5%) | 3/37 (8%) | 2/5 (40%) | 3/51 (6%) | 1/30 (3%) | 5/42 (12%) | 4/81 (5%) | 0.27 |  |
| **Postoperative Time to Mortality (days)** | 12 (5-44) | 9 (4-42) | 15 (9-17) | 15 (10-20) | 139 (133-202) | 162 | 15 (5-19) | 151 (136-188) | 0.02 |  |
