# Supplementary material for: Primary vs Staged Biventricular Repair for Neonatal IAA with VSD and LVOTO
Source: Ann Thorac Surg Short Rep. 2024 May 22;2(4):815–9. doi: 10.1016/j.atssr.2024.04.025 (PMC11708735; doi:10.1016/j.atssr.2024.04.025)
Supplement: Supplementary Table 2 [file mmc3.docx]

| **Supplemental Table 2. Second Stage Biventricular Conversion Operations for IAA/VSD with LVOTO** | | | |
| --- | --- | --- | --- |
|  | **Patients with History of Norwood** | **Patients with History of Hybrid Stage I** | **All Patients Undergoing Second Stage Biventricular Conversion** |
|  |  |  |  |
| **Number of Patients** | 51 | 7 | 58 |
| **Gestation (weeks)** | 37.7 (1.8) | 38.3 (2.0) | 37.8 (1.8) |
| **Prematurity (n/N, %)** | 10/51 (20%) | 2/7 (29%) | 12/58 (21%) |
| **Birth Weight (kg)** | 2.88 (0.59) | 2.85 (0.43) | 2.88 (0.56) |
| **Male (n/N, %)** | 25/51 (49%) | 1/7 (14%) | 26/58 (45%) |
| **White/Caucasian (n/N, %)** | 24/49 (49%) | 6/7 (86%) | 30/56 (54%) |
| **Preoperative Factors** |  |  |  |
| Shock, ongoing | 0/51 (0%) | 0/7 (0%) | 0/58 (0%) |
| Shock, resolved | 1/51 (2%) | 0/7 (0%) | 1/58 (2%) |
| Taking steroids | 1/51 (2%) | 0/7 (0%) | 1/58 (2%) |
| Hypocoagulable | 2/51 (4%) | 2/7 (29%) | 4/58 (7%) |
| Renal Dysfunction | 1/51 (2%) | 0/7 (0%) | 1/58 (2%) |
| Renal Failure, dialysis | 0/51 (0%) | 0/7 (0%) | 0/58 (0%) |
| Mechanical Ventilation | 2/51 (4%) | 2/7 (29%) | 4/58 (7%) |
| Any | 30/51 (59%) | 6/7 (86%) | 36/58 (62%) |
| **Syndromes** |  |  |  |
| DiGeorge | 24/44 (55%) | 1/5 (20%) | 25/49 (51%) |
| Fetal Alcohol Syn or Drug Exposure | 1/44 (2%) | 0/5 (0%) | 1/49 (2%) |
| Any | 28/44 (64%) | 2/5 (40%) | 30/49 (61%) |
| **Chromosomal Abnormalities** |  |  |  |
| 22q11 | 24/44 (55%) | 1/5 (20%) | 25/49 (51%) |
| Any | 27/44 (61%) | 4/5 (80%) | 31/49 (63%) |
| **Non-Cardiac Abnormalities** |  |  |  |
| Airway Issues | 8/42 (19%) | 1/5 (20%) | 9/47 (19%) |
| Any | 12/42 (29%) | 2/5 (40%) | 14/47 (30%) |
| **Surgery Performed** |  |  |  |
| Rastelli | 31/51 (61%) | 1/7 (14%) | 32/58 (55%) |
| Biventricular Conversion | 12/51 (24%) | 0/7 (0%) | 12/58 (21%) |
| Yasui | 5/51 (10%) | 6/7 (86%) | 11/58 (19%) |
| Ross/Ross-Konno | 3/51 (6%) | 0/7 (0%) | 3/58 (5%) |
| **Perioperative Details** |  |  |  |
| Age (days) | 278 (227-375) | 187 (152-208) | 265 (217-356) |
| Weight (kg) | 7.7 (6.9-9.3) | 6.9 (6.1-7) | 7.6 (6.8-9.1) |
| Operative Time (min) | 339 (272-441) | 440 (398-458) | 349 (293-454) |
| CPB Time (min) | 169 (144-223) | 235 (217-272) | 177 (145-223) |
| Cross Clamp Time (min) | 108 (90-143) | 134 (103-139) | 110 (90-143) |
| **Complications** |  |  |  |
| Any Major Complication | 19/51 (37%) | 4/7 (57%) | 23/58 (40%) |
| Median Number of Major Complications per Patient | 0 (0-1) | 1 (0-1.5) | 0 (0-1) |
| Ventilator > 7 days | 11/51 (22%) | 2/7 (29%) | 13/58 (22%) |
| Unplanned Reintubation | 9/51 (18%) | 3/7 (43%) | 12/58 (21%) |
| Recurrent Laryngeal Nerve Injury | 1/51 (2%) | 1/7 (14%) | 2/58 (3%) |
| Any Complication | 41/51 (80%) | 5/7 (71%) | 46/58 (79%) |
| Median Number of Complications per Patient | 2 (1-3.5) | 4 (2-5.5) | 2 (1-4) |
| **Length of Stay (days)** | 16 (9-32) | 50 (15-78) | 16 (9-42) |
| **Postoperative Length of Stay (days)** | 14 (8-24) | 45 (15-70) | 15 (8-28) |
| **Operative Mortality** | 0/51 (0%) | 1/7 (14%) | 1/58 (2%) |
| **Postoperative Time to Mortality (days)** | N/a | 44 | 44 |
